# A replication study of the association between the *IL12B *promoter allele CTCTAA and susceptibility to cerebral malaria in Thai population

**DOI:** 10.1186/1475-2875-8-290

**Published:** 2009-12-11

**Authors:** Izumi Naka, Jintana Patarapotikul, Katsushi Tokunaga, Hathairad Hananantachai, Naoyuki Tsuchiya, Jun Ohashi

**Affiliations:** 1 Doctoral Program in Life System Medical Sciences, Graduate School of Comprehensive Human Sciences, University of Tsukuba, Ibaraki, Japan; 2 Department of Microbiology & Immunology, Faculty of Tropical Medicine, Mahidol University, Bangkok, Thailand; 3 Department of Human Genetics, Graduate School of Medicine, The University of Tokyo, Tokyo, Japan

## Abstract

**Background:**

Interleukin-12 (IL-12), a heterodimeric cytokine composed of p35 and p40 subunits, has been thought to play an important role in the pathogenesis of malaria. The IL-12p40 subunit is encoded by the *IL12B *gene. An *IL12B *promoter allele, CTCTAA, at rs17860508 has been reported to be associated with susceptibility to cerebral malaria in African populations. However, this association has not so far been replicated in non-African populations.

**Methods:**

To examine whether the CTCTAA allele is associated with susceptibility to cerebral malaria in Asian populations, 303 Thai patients with *Plasmodium falciparum *malaria (109 cerebral malaria and 194 mild malaria patients) were genotyped for rs17860508 by PCR-direct sequencing.

**Results:**

The CTCTAA allele showed a significant association with susceptibility to cerebral malaria in the Thai population (allelic OR = 1.37; one sided *P*-value = 0.030).

**Conclusions:**

The existence of a significant association between the CTCTAA allele and susceptibility to cerebral malaria was confirmed in Southeast Asian population, which was previously reported in African populations.

## Background

Cerebral malaria, a severe form of *Plasmodium falciparum *malaria, causes the vast majority of deaths related to *P. falciparum *infection. Although the pathophysiology of severe malaria (including cerebral malaria) is not fully understood, significant differences in serum or plasma concentrations of various cytokines have been observed between severe and mild malaria patients. The levels of interleukin-12 (IL-12) are significantly lower in patients with severe malaria in comparison to those with mild malaria [1,2]. In animal models of malaria, IL-12 has been shown to have a protective role [3-5], although pathogenic effects of IL-12 in malaria have also been reported [5]. Considering the fact that only some malaria patients develop cerebral malaria, human genetic polymorphisms may play an important role in determining the severity of the disease. In this context, genes encoding IL-12 are attractive candidates for determining the susceptibility to cerebral malaria.

Interleukin-12 (IL-12) is a heterodimeric cytokine formed by a 35-kDa light chain (p35 or IL-12a) and a 40-kDa heavy chain (p40 or IL-12b). The IL-12p40 heavy chain is encoded by the *IL12B *gene. The *IL12B *has a functional polymorphism, CTCTAA/GC (rs17860508), with a 4 bp difference between the two alleles in the promoter region. The CTCTAA/GC polymorphism has been shown to affect the gene expression and the production of cytokines and nitric oxide [6-9]. The homozygotes of CTCTAA were reported to be associated with increased mortality in Tanzanian children with cerebral malaria [9]. Recently, the strong association of CTCTAA with the risk of cerebral malaria was also observed in family-based association studies in Mali [10]. However, this association has not yet been examined in non-African populations, such as Southeast Asians. The purpose of this study is to assess whether the *IL12B *promoter allele, CTCTAA, is associated with susceptibility to cerebral malaria in Thais.

## Methods

### Subjects

A total of 303 adult patients with *P. falciparum *malaria (109 cerebral malaria and 194 mild malaria patients) living in northwest Thailand were analysed in this study. All patients underwent treatment at the Hospital for Tropical Diseases, Faculty of Tropical Medicine, Mahidol University. Malarial infection by *P. falciparum *was confirmed in all patients by a positive blood smear for the asexual form of *P. falciparum*. Clinical manifestations of malaria were classified according to the definitions and associated criteria by the World Health Organization. Cerebral malaria was defined as unrousable coma, a positive result in tests for the presence of an asexual form of *P. falciparum*, and exclusion of other causes of coma. Mild malaria was defined as having a positive blood smear and fever without other causes of infection and no signs indicating severe malaria such as high parasitaemia (> 100,000 parasites/ml), hypoglycemia (glucose level < 22 nmol/L), severe anaemia (haematocrit < 20% or hemoglobin level < 7.0 g/dl) or increased serum level of creatinine (> 3.0 mg/dl). All individuals were 13 years old or older, and the mean ages of patients with mild malaria and cerebral malaria patients were 25.5 and 28.6 respectively. This study was approved by the institutional review board of the Faculty of Tropical Medicine, Mahidol University, and the Research Ethics Committee of the Graduate School of Comprehensive Human Sciences, University of Tsukuba. Informed consent was obtained from all patients.

### Genotyping

Genomic DNA was extracted from peripheral blood leukocytes using a QIAamp Blood Kit (Qiagen, Hilden, Germany). The *IL12B *promoter polymorphism, rs17860508, was genotyped by PCR-direct sequencing with an ABI PRISMTM 3100 Genetic Analyzer (Perkin-Elmer Applied Biosystems). PCR was performed using a primer set: a 5' primer IL12B-proF: 5'-GGATCAGGGTCTGGATTGTG-3' and a 3' primer IL12B-proR: 5'-GGCTGATGCTTGGAGATTGT-3'. The amplification condition consisted of an initial denaturation at 96°C for 10 min, followed by 35 cycles of denaturation at 96°C for 30 sec, annealing at 60°C for 30 sec, and extension at 72°C for 1 min using a thermal cycler (GeneAmp PCR system 9700; Perkin-Elmer Applied Biosystems).

### Statistical analysis

The differences in genotype and allele frequencies between the patients with cerebral and mild malaria were examined by the Cochran-Armitage test for trend and chi-square test, respectively. The *P*-values for association were calculated by a one-sided test to avoid a false negative because the CTCTAA allele had been previously reported to be associated with both susceptibility to and mortality from cerebral malaria. The deviation from Hardy-Weinberg equilibrium (HWE) for each malaria group was assessed by a chi-square test. A *P*-value of less than 0.05 was regarded as statistically significant in this study.

## Results and Discussion

The genotype of rs17860508 was analysed in 303 Thai patients with malaria (Table 1). The genotype frequency did not deviate from HWE in each malaria group (*P*-value > 0.05). Consistent with observations in African populations, the CTCTAA allele showed a significant association with susceptibility to cerebral malaria in the Thai population (allelic OR = 1.37; one sided *P*-value = 0.030). The Cochran-Armitage trend test for the genotype frequency also established the association between the CTCTAA allele and cerebral malaria (*P*-value = 0.038). Although a previous study demonstrated that the CTCTAA/GC heterozygotes increased the risk of cerebral malaria [10], such a tendency was not observed in the present study. In Thais, the risk of cerebral malaria seems to correlate with the number of copies of the CTCTAA allele (i.e., 0, 1, or 2) in malaria patients. This may be the ethnic difference between African and Asian.

**Table 1:** Genotype and allele frequencies of the *IL12B *promoter polymorphism in Thai malaria patients.

	Frequency		*P*-value
		
	Cerebral malaria (n = 109)	Mild malaria (n = 194)	
Genotype:			
CTCTAA/CTCTAA	34 (0.312)	49 (0.252)	0.038^a^
CTCTAA/GC	51 (0.468)	83 (0.428)	
GC/GC	24 (0.220)	62 (0.320)	
			
Allele:			
CTCTAA	119 (0.546)	181 (0.466)	0.030^b^
GC	99 (0.454)	207 (0.534)	

^a^One-sided *P*-value was calculated based on the result from the Cochran-Armitage test for trend.

^b^One-sided *P*-value was calculated based on the result from chi-square test.

The significant association of the CTCTAA allele with susceptibility to cerebral malaria was found both in children living in Mali [10] and in Thai adults (this study). In areas of low endemicity, where the immunity is low, severe infection occurs in all age groups including adults as in the studied population. Since the endemicity differs between Africa and Southeast Asia, it should not readily be concluded that the present association of the CTCTAA allele with susceptibility to cerebral malaria is independent of age. To evaluate this, further association studies are required to be conducted in Thai children.

The present results suggest that the *IL12B *promoter polymorphism at rs17860508 plays an important role in determining the onset of cerebral malaria, although the role of IL-12 in malaria infection remains to be studied.

## Conclusions

A significant association of the *IL12B *promoter CTCTAA allele with cerebral malaria was replicated in a Thai population.

## Competing interests

The authors declare that they have no competing interests.

## Authors' contributions

NI performed the genotyping, helped conduct statistical analyses, and wrote the manuscript. KT helped genotype the samples. JP and HH collected blood samples and extracted DNA. JP and JO participated in the design and coordination of the study. NT was involved in the interpretation of the data and preparation of the manuscript. JO performed statistical analyses and helped write the manuscript. All authors read and approved the final manuscript.

## References

[B1] Luty AJ Perkins DJ Lell B Schmidt-Ott R Lehman LG Luckner D Greve B Matousek P Herbich K Schmid D Weinberg JB Kremsner PG Low interleukin-12 activity in severe Plasmodium falciparum malaria Infect Immun 2000 68 3909 3915 10.1128/IAI.68.7.3909-3915.2000 10858202 PMC101666

[B2] Perkins DJ Weinberg JB Kremsner PG Reduced interleukin-12 and transforming growth factor-beta1 in severe childhood malaria: relationship of cytokine balance with disease severity J Infect Dis 2000 182 988 992 10.1086/315762 10950804

[B3] Crutcher JM Stevenson MM Sedegah M Hoffman SL Interleukin-12 and malaria Res Immunol 1995 146 552 559 10.1016/0923-2494(96)83031-8 8839161

[B4] Mohan K Sam H Stevenson MM Therapy with a combination of low doses of interleukin 12 and chloroquine completely cures blood-stage malaria, prevents severe anemia, and induces immunity to reinfection Infect Immun 1999 67 513 519 9916053 10.1128/iai.67.2.513-519.1999 PMC96349

[B5] Stevenson MM Su Z Sam H Mohan K Modulation of host responses to blood-stage malaria by interleukin-12: from therapy to adjuvant activity Microbes Infect 2001 3 49 59 10.1016/S1286-4579(00)01354-X 11226854

[B6] Morahan G Huang D Wu M Holt BJ White GP Kendall GE Sly PD Holt PG Association of IL12B promoter polymorphism with severity of atopic and non-atopic asthma in children Lancet 2002 360 455 459 10.1016/S0140-6736(02)09676-9 12241719

[B7] Muller-Berghaus J Kern K Paschen A Nguyen XD Kluter H Morahan G Schadendorf D Deficient IL-12p70 secretion by dendritic cells based on IL12B promoter genotype Genes Immun 2004 5 431 434 10.1038/sj.gene.6364102 15175646

[B8] Peng JC Abu Bakar S Richardson MM Jonsson JJ Frazer IH Nielsen LK Morahan G Thomas R IL10 and IL12B polymorphisms each influence IL-12p70 secretion by dendritic cells in response to LPS Immunol Cell Biol 2006 84 227 232 10.1111/j.1440-1711.2006.01419.x 16519741

[B9] Morahan G Boutlis CS Huang D Pain A Saunders JR Hobbs MR Granger DL Weinberg JB Peshu N Mwaikambo ED Marsh K Roberts DJ Anstey NM A promoter polymorphism in the gene encoding interleukin-12 p40 (IL12B) is associated with mortality from cerebral malaria and with reduced nitric oxide production Genes Immun 2002 3 414 418 10.1038/sj.gene.6363909 12424623

[B10] Marquet S Doumbo O Cabantous S Poudiougou B Argiro L Safeukui I Konate S Sissoko S Chevereau E Traore A Keita MM Chevillard C Abel L Dessein AJ A functional promoter variant in IL12B predisposes to cerebral malaria Hum Mol Genet 2008 17 2190 2195 10.1093/hmg/ddn118 18413324

